# Prostatic Urethral Lift for Obstructive Median Lobes: Consistent Results Across Controlled Trial and Real-World Settings

**DOI:** 10.1089/end.2022.0324

**Published:** 2022-12-27

**Authors:** Gregg Eure, Daniel Rukstalis, Claus Roehrborn

**Affiliations:** ^1^ Department of Urology, Urology of Virginia, Virginia Beach, Virginia, USA.; ^2^ Prisma Health USC Medical Group, Division of Urology, 300 Palmetto Health Pkwy, Columbia.; ^3^ Department of Urology, UT Southwestern, Dallas, Texas, USA.

**Keywords:** lower urinary tract symptoms, retrospective study, real world, prostatic urethral lift, benign prostatic hyperplasia, randomized controlled trials, clinically controlled trials, CCT, minimally invasive surgical therapy, transurethral resection of the prostate, symptom score, IPSS

## Abstract

**Introduction:**

The evidence for prostatic urethral lift (PUL), in treating lower urinary tract symptoms/benign prostatic hyperplasia (BPH) in men with obstructive median lobes (OMLs), has grown. In this study, we present the first detailed comparison of outcomes between OML patients treated with PUL in controlled and real-world settings to relevant comparators (subjects treated with transurethral resection of the prostate [TURP] and sham in randomized controlled trials [RCTs]) to demonstrate similar symptom, safety, and patient experience outcomes.

**Materials and Methods:**

Symptom and safety outcomes and patient satisfaction were compared through 12 months among controlled PUL studies: BPH6 RCT (35 men randomized to TURP); L.I.F.T. pivotal RCT in subjects with lateral lobe obstruction (66 subjects randomized to sham) and MedLift, an U.S. Food and Drug Administration-approved Investigational Device Exemption (IDE) extension of the L.I.F.T. trial (45 men with OML). Symptom improvement, catheterization, and adverse event rates were compared between MedLift subjects and OML patients (*n* = 187) from the large real-world retrospective (RWR) study of PUL filtered on baseline characteristics to approximate the MedLift population.

**Results:**

Posttreatment, International Prostate Symptoms Score (IPSS) improvement for MedLift subjects was 170% greater compared with sham at 3 months with significantly better quality of life (QoL), Qmax, and benign prostatic hyperplasia impact index (BPHII). Compared with TURP, MedLift IPSS and QoL improved significantly better at 1 and 3 months and with superior ejaculatory function scores at all time points after PUL. IPSS, QoL, postvoid residual (PVR), and Qmax outcomes were equivalent between MedLift and RWR OML groups at 3, 6, and 12 months. RWR OML patients did not experience higher rates of overall adverse events compared with MedLift.

**Conclusion:**

Controlled and real-world outcomes confirm PUL is a safe and effective treatment for BPH patients with and without OML.

## Introduction

Since its approval by the U.S. Food and Drug Administration (FDA) in 2013, prostatic urethral lift (PUL) for the treatment of bothersome lower urinary tract symptoms attributed to benign prostatic hyperplasia (BPH) has experienced a growth in adoption.^1,2^ Today hundreds of thousands of men have been treated with PUL and it is considered a standard of care in the United States and abroad, with gland morphology and size assessment an important component of the initial evaluation.^3–6^ The procedure relieves urinary flow obstruction and provides patients with rapid, durable relief.^7,8^ The rise of PUL has once again positioned minimally invasive surgical therapy (MIST) as an attractive option for patients unsuccessfully treated by medical therapy or with poor compliance, yet hesitant to undergo traditional surgery, a sentiment acknowledged by the European Association of Urology (EAU) and the American Urological Association (AUA).^6,9^

The FDA indicates PUL for the treatment of BPH, including lateral and median lobe hyperplasia, in men with prostates no greater than 100 cc. This indication is based on review of randomized controlled trial (RCT), controlled clinical trial (CCT), and single-arm trial studies. Society guidelines, however, often provide recommendations that are narrowed, due to their more limited evaluation of published evidence. For instance, the AUA BPH guidelines state that only RCT and CCT studies are considered when crafting evidence-based updates, and thus limit PUL recommendation to only lateral lobe (LL) disease with a maximum prostate volume of 80 cc. The EAU BPH guidelines give a strong recommendation for PUL but limit the maximum volume to 70 cc and also exclude middle lobe.

Because the regulatory/legal indication for PUL is broader than the current academic guidelines, there has been extensive use of PUL to treat men with prostates outside of the guideline volume limit and with obstructive median lobe (OML). The MedLift study, an FDA-approved IDE extension of the L.I.F.T. pivotal trial, was the first study to compare PUL outcomes for OML to those for LL and concluded that PUL OML treatment was not inferior to LL treatment.^10^ MedLift enrolled subjects with the same criteria as the L.I.F.T. trial (which randomized LL patients to either PUL or sham treatment), however patients presented with an ultrasound-defined OML. Utilizing the sham subjects from L.I.F.T. as controls for PUL OML treatment thus positions MedLift as a CCT. Within this study, we compare outcomes of OML patients treated with PUL in controlled and real-world settings to relevant comparator groups (i.e., subjects treated with transurethral resection of the prostate [TURP] and sham in RCT) to demonstrate similar symptom, safety, and patient experience outcomes.

## Materials and Methods

### Study protocol

Details of the four PUL studies used in this analysis have been published and are summarized in Table 1. The study protocols were in accordance with all applicable U.S. Federal and state laws and regulations, including 45 CFR 46 and the HIPAA Privacy Rule.

**Table 1.** Design Details of Prostatic Urethral Lift Clinical Studies and Subjects Used for the Comparative Analysis

Clinical study	Type of study	Subjects used in comparative analysis	Outcomes measures
L.I.F.T.	RCT PUL+Sham control	66 men randomized to sham control (rigid cystoscopy) ≥50 years old, IPSS ≥13, Qmax ≤12 mL/s, prostate volume 30–80 cm^3^	IPSS, PVR, Qmax, QoL, MSHQ Bother, MSHQ EJD, BPHII, SHIM
BPH6	RCT PUL+TURP	35 men randomized to TURP ≥50 years old, IPSS ≥13, Qmax ≤15 mL/s, prostate volume <60 cm^3^	IPSS, PVR, Qmax, QoL, MSHQ Bother, MSHQ EJD, BPHII, SHIM
MedLift	CCT Single arm; PUL in subjects w/OML	45 men who met the inclusion criteria of the L.I.F.T. study and had an OML	IPSS, PVR, Qmax, QoL, MSHQ Bother, MSHQ EJD, BPHII, SHIM
RWR	Large, retrospective database; consecutive PUL patients after market clearance	2078 patients not in retention at baseline, ≥8 baseline IPSS and w/o prior BPH treatment Filtered to match MedLift and stratified by obstructive morphology (OML, *n* = 180; LL, *n* = 1271)	IPSS, PVR, Qmax, QoL

BPH = benign prostatic hyperplasia; BPHII = benign prostatic hyperplasia impact index; CCT = Controlled Clinical Trial; EJD = ejaculatory dysfunction; IPSS = International Prostate Symptoms Score; LL = lateral lobe; MSHQ = Male Sexual Health Questionnaire; OML = obstructive median lobe; PUL = prostatic urethral lift; PVR = postvoid residual; QoL = quality of life; RCT = randomized controlled trial; RWR = real-world retrospective; SHIM = Sexual Health Inventory for Men; TURP = transurethral resection of the prostate.

### Study procedures

**FIG. 1.**
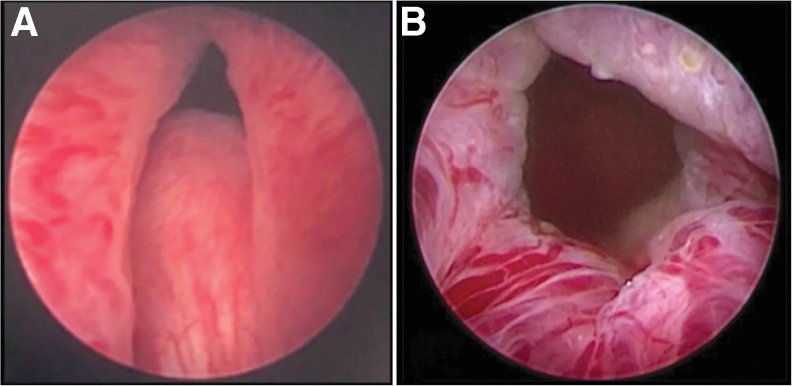
Cystoscopic evaluation of median lobe obstruction **(A)** before and **(B)** after treatment with the PUL procedure utilizing the UroLift System. PUL = prostatic urethral lift.

During the PUL procedure, small, transprostatic implants (UroLift System; NeoTract, Inc./Teleflex, Pleasanton, CA) are deployed under cystoscopic guidance, mechanically retracting obstructive prostatic lobes, and creating an anterior channel through the prostatic fossa (Fig. 1). In patients with OMLs, the base of the tissue protruding intravesicularly is retracted distally into the prostatic fossa and secured to either side of the urethra using the implant, thereby extending the channel to the bladder neck through displacement of the middle lobe as described in the MedLift CCT.

In the pivotal L.I.F.T. study, a sham control procedure was conducted to mimic the PUL experience as closely as possible: a surgical drape was used as a visual partition and a rigid cystoscopy performed, with the surgeon calling for devices that were opened but not deployed. A disposable biopsy device was deployed to simulate device sounds. TURP control procedures in the BPH6 RCT were conducted by experienced urologists in accordance with their standard operating procedure.

For the Real-World Retrospective (RWR) Study of PUL, retrospective chart review of PUL procedures was conducted across 22 sites in the United States, Australia, and the United Kingdom between July 2017 and March 2020. Consecutive PUL cases following market clearance were included in the database totaling a number of 3226 patients. The presence of an obstructing median lobe was based on physician cystoscopic assessment during the procedure.

### Comparative study assessments

#### MedLift CCT PUL outcomes *vs* RCT controls

The following MedLift CCT (*n* = 45) outcomes were compared with those of RCT control procedures (sham: *n* = 66; TURP: *n* = 35) at 1, 3, 6, 12, and 24 months: International Prostate Symptoms Score (IPSS), quality of life (QoL), Qmax, postvoid residual (PVR), patient satisfaction (rating postprocedural condition as very much better, much better, a little better, no change, a little worse, much worse, or very much worse), catheterization, surgical retreatment, adverse events (including serious adverse events), and sexual function (Benign Prostatic Hyperplasia Impact Index, Sexual Health Inventory for Men, Men's Sexual Health Questionnaire Ejaculatory Dysfunction/Bother). Mean change and percent change from baseline were compared using *t*-tests and 95% confidence intervals (CI); unpaired *t*-tests were used to compare absolute symptom scores and Fisher's exact test was performed to assess adverse event and catheterization rates. It should be noted that the sham and TURP arm patients did not have median lobes, however. Standard deviations between MedLift and BPH6 were reviewed and determined to provide statistical weight in pairwise comparison to permit appropriate statistical comparisons.

#### MedLift CCT outcomes *vs* RWR study

MedLift results were compared with those of two subgroups in the RWR study: RWR OML and RWR LL patients. After filtering for baseline IPSS, Qmax, PVR, prostate volume, and no previous BPH procedures to mirror MedLift and L.I.F.T. enrollment criteria, 180 of 244 OML and 1279 of 1834 LL total RWR subjects were used in the comparative assessments, respectively. Effectiveness was evaluated by comparing IPSS, QoL, Qmax, and PVR at baseline and at 1-, 3-, 6-, 12-, and 24-month post-PUL. Mean change and percentage change from baseline were compared using paired *t*-tests and 95% CIs. Absolute symptom scores were compared between groups using unpaired *t*-test; adverse event and catheterization rates were analyzed using chi-squared and Fisher's exact test when appropriate. Analyzed cohorts were statistically powered to allow for comparative analysis.

## Results

### PUL for OML in controlled trials

**FIG. 2.**
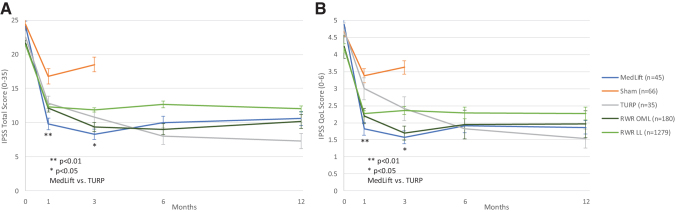
**(A)** IPSS and **(B)** QoL response following treatment with PUL, sham, and TURP across controlled and real-world studies for PUL. IPSS = International Prostate Symptoms Score; TURP = transurethral resection of the prostate; QoL = quality of life.

**FIG. 3.**
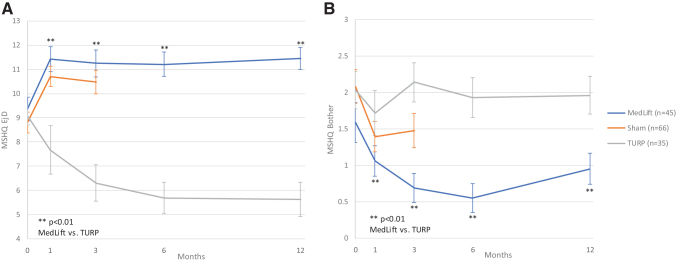
Ejaculatory function **(A)** and Bother **(B)** scores following treatment with PUL, sham, and TURP in controlled studies of PUL.

**FIG. 4.**
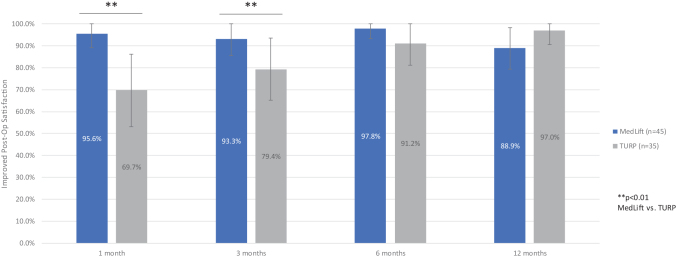
Postprocedural satisfaction scores following treatment with PUL or TURP in controlled studies of PUL.

Baseline demographics were similar in all compared groups (Table 2). Statistical difference was only seen for baseline Qmax in TURP control and MedLift subjects (Table 2). At 3 months, MedLift subjects experienced 170% greater IPSS improvement than sham control subjects and significantly better QoL, Qmax, and benign prostatic hyperplasia impact index (BPHII) outcomes (Table 3 and Fig. 2A,B). MedLift IPSS and QoL were significantly improved compared with TURP controls at 1 and 3 months postprocedure and were equivalent at 6 and 12 months. There were no differences in percent change in Qmax and BPHII and change in PVR and Sexual Health Inventory for Men (SHIM) scores between MedLift and TURP at 12 months posttreatment (Tables 3 and 5). MedLift ejaculatory function scores were significantly better than TURP at all time points after PUL (Table 5 and Fig. 3A,B). A significantly higher percentage of MedLift patients described their postprocedural condition as ‘better than baseline’ *vs* TURP after 1 and 3 months, which was then similar at 6 and 12 months postprocedure (Fig. 4).

**Table 2.** Baseline Demographics of Patients Used for the Comparative Analysis

Mean (SD) [range]	MedLift CCT (*n* = 45)	Sham control (L.I.F.T. RCT) (*n* = 66)	TURP control (BPH6 RCT) (*n* = 35)	RWR OML (*n* = 180)	RWR LL (*n* = 1279)
Age (years)	63.93 (6.96) [51.0–80.0]	64.55 (8.04) [50.0–84.0]	65.4 (6.35) [51.0–78.0]	69.4 (8.6) [46–94]	68.8 (9.2) [30–96]
Body mass index	28.8 (3.56) [22.3–38.0]	28.25 (6.27) [19.5–55.7]	—	28.8 (5.5) [18–46]	29.0 (5.6) [15–75]
Prostate-specific antigen (ng/mL)	2.48 (1.86) [0.4–9.9]	2.08 (1.63) [0.3–7.1]	2.59 (2.11) [0.3–8.6]	2.4 (2.3) [0–19]	2.5 (3.3) [0–59]
Prostate volume (cm^3^)	44.22 (11.18) [30.4–68.4]	40.93 (10.8) [30.0–75.5]	40.66 (13.4) [17.0–68.4]	52.9 (21.1) [19–166]	43.5 (15.7) [12–80]
IPSS	24.16 (4.95) [13.0–35.0]	24.41 (5.75) [13.0–33.0]	22.51 (5.87) [13.0–34.0]	20.5 (6) [8–35]	21.3 (5.2) [13–35]
BPHII	7.69 (2.79) [2.0–12.0]	7.02 (3.03) [0.0–13.0]	7.21 (3.03) [1.0–12.0]	—	—
QoL	4.89 (0.8) [3.0–6.0]	4.67 (1.1) [2.0–6.0]	4.71 (1.23) [2.0–6.0]	4.0 (1.3) [0–6]	4.1 (1.2) [0–6]
PVR (mL)	107.3 (79.94) [0.0–291]	87.73 (72.36) [0.0–244]	101.7 (86.87) [0.0–328]	104.1 (117.6) [0–599]	71.2 (62.2) [0–250]
Qmax (mL/s)	7.16 (2.85) [1.0–12.0]	7.88 (2.39) [2.0–12.0]	9.53 (3.21) [3.0–15.0]	9.6 (4.3) [1–25]	8.4 (2.4) [1–12]

**Table 3.** RCT and CCT Outcomes over 12 Months After Treatment with Prostatic Urethral Lift, Sham, and Transurethral Resection of the Prostate

	Baseline				1 Month				3 Months				6 Months				12 Months			
	MedLift	L.I.F.T.–LL	L.I.F.T.–sham	BPH–TURP	MedLift	L.I.F.T.–LL	L.I.F.T.–sham	BPH–TURP	MedLift	L.I.F.T.–LL	L.I.F.T.–sham	BPH–TURP	MedLift	L.I.F.T.–LL	L.I.F.T.–sham	BPH–TURP	MedLift	L.I.F.T.–LL	L.I.F.T.–sham	BPH–TURP
IPSS *N* (paired)	45	140	66	35	45	135	66	33	45	136	66	34	45	133	20	33	44	123	11	32
Baseline (SD)	24.16 ± 4.95	22.2 ± 5.5	24.41 ± 5.75	22.51 ± 5.87	24.16 ± 4.95	22.27 ± 5.49	24.41 ± 5.75	22.85 ± 5.80	24.16 ± 4.95	22.31 ± 5.49	24.41 ± 5.75	22.56 ± 5.96	24.16 ± 4.95	22.21 ± 5.51	21.45 ± 5.85	22.61 ± 6.04	24.11 ± 5.00	22.13 ± 5.56	20.73 ± 5.48	22.78 ± 5.88
Follow-up (SD)	—	—	—	—	9.80 ± 5.70	12.28 ± 6.94	16.82 ± 9.09	12.88 ± 5.87	8.27 ± 5.12	11.17 ± 7.68	18.53 ± 8.59	10.76 ± 8.40	9.96 ± 6.39	11.24 ± 7.31	17.00 ± 7.75	8.03 ± 7.15	10.64 ± 6.96	11.52 ± 7.27	12.73 ± 9.31	7.34 ± 6.32
Change (SD)	0.00 ± 0.00	0.00 ± 0.00	0.00 ± 0.00	0.00 ± 0.00	−14.4 ± 6.73	−9.99	−7.59 ± 8.53	−9.97 ± 7.92	−15.9 ± 6.77	−11.14	−5.88 ± 7.65	−11.8 ± 9.46	−14.2 ± 7.61	−10.97	−4.45 ± 7.18	−14.6 ± 8.51	−13.5 ± 7.74	−10.61	−8.00 ± 9.00	−15.4 ± 6.83
Change *vs* MedLift *p*-value	—	—	—	—	—	<0.01	<0.01	0.01	—	<0.01	<0.01	0.03	—	0.01	<0.01	0.84	—	0.03	0.04	0.26
QoL *N* (paired)	45	140	66	35	45	135	66	33	45	136	66	34	45	133	20	33	44	123	11	32
Baseline (SD)	4.89 ± 0.80	4.6 ± 1.1	4.67 ± 1.10	4.71 ± 1.23	4.89 ± 0.80	4.61 ± 1.06	4.67 ± 1.10	4.79 ± 1.22	4.89 ± 0.80	4.62 ± 1.06	4.67 ± 1.10	4.76 ± 1.21	4.89 ± 0.80	4.60 ± 1.06	4.40 ± 0.99	4.73 ± 1.21	4.89 ± 0.81	4.56 ± 1.01	4.09 ± 1.04	4.63 ± 1.24
Follow-up (SD)	—	—	—	—	1.82 ± 1.21	2.59 ± 1.68	3.38 ± 1.62	3.00 ± 1.85	1.58 ± 1.27	2.40 ± 1.72	3.62 ± 1.61	2.41 ± 2.02	1.91 ± 1.38	2.17 ± 1.65	3.75 ± 1.21	1.82 ± 1.72	1.86 ± 1.32	2.25 ± 1.61	2.55 ± 1.13	1.53 ± 1.54
Change (SD)	0.00 ± 0.00	0.00 ± 0.00	0.00 ± 0.00	0.00 ± 0.00	−3.07 ± 1.50	−2.02	−1.29 ± 1.58	−1.79 ± 1.95	−3.31 ± 1.52	−2.22	−1.05 ± 1.49	−2.35 ± 2.00	−2.98 ± 1.63	−2.44	−0.65 ± 0.99	−2.91 ± 1.88	−3.02 ± 1.53	−2.31	−1.55 ± 1.37	−3.09 ± 1.55
Change *vs* MedLift *p*-value	—	—	—	—	—	<0.01	<0.01	<0.01	—	<0.01	<0.01	0.02	—	0.06	<0.01	0.86	—	0.01	<0.01	0.84
PVR *N* (paired)	45	140	66	35	44	—	—	—	45	136	65	32	45	—	—	31	44	120	9	32
Baseline (SD)	107.3 ± 79.94	85.5 ± 69.2	87.73 ± 72.36	101.7 ± 86.87	109.3 ± 79.78	—	—	—	107.3 ± 79.94	85.01 ± 68.6	85.62 ± 70.84	98.56 ± 84.95	107.3 ± 79.94	—	—	100.5 ± 85.67	108.5 ± 80.48	84.54 ± 66.11	82.89 ± 80.17	103.5 ± 89.75
Follow-up (SD)	—	—	—	—	73.57 ± 62.83	—	—	—	77.82 ± 67.30	76.07 ± 83.10	63.40 ± 63.99	47.59 ± 48.72	75.62 ± 73.28	—	—	46.23 ± 49.07	69.93 ± 77.04	72.43 ± 99.85	73.56 ± 78.40	33.56 ± 38.62
Change (SD)	0.00 ± 0.00	0.00 ± 0.00	0.00 ± 0.00	0.00 ± 0.00	−35.7 ± 87.36	—	—	—	−29.5 ± 81.77	−9.01 ± 85.71	−22.2 ± 70.69	−51.0 ± 78.72	−31.7 ± 73.28	—	—	−54.2 ± 84.63	−38.6 ± 84.61	−12.11 ± 100.39	−9.33 ± 62.27	−70.0 ± 78.96
Change *vs* MedLift *p*-value	—		—	—	—	—	—	—	—	0.02	0.62	0.25	—	—	—	0.22	—	0.004	0.33	0.10

RCT = randomized controlled trial; CCT = controlled clinical trial.

**Table 4.** Real-World Outcomes over 12 Months Following Treatment with Prostatic Urethral Lift

	Baseline		1 Month		3 Months		6 Months		12 Months	
	RWR OML	RWR LL	RWR OML	RWR LL	RWR OML	RWR LL	RWR OML	RWR LL	RWR OML	RWR LL
IPSS *N* (paired)	180	1279	120	820	79	576	30	239	30	241
Baseline (SD)	21.63 ± 5.27	21.34 ± 5.22	21.50 ± 5.34	21.11 ± 5.18	22.01 ± 5.33	21.20 ± 5.10	20.33 ± 5.07	21.74 ± 5.51	21.77 ± 7.02	20.49 ± 5.25
Follow-up (SD)	—	—	12.10 ± 6.27	12.34 ± 7.09	9.38 ± 5.80	11.86 ± 7.22	9.02 ± 4.38	12.68 ± 7.78	10.18 ± 5.70	12.01 ± 7.36
Change (SD)	0.00 ± 0.00	0.00 ± 0.00	−9.40 ± 7.77	−8.78 ± 8.05	−12.6 ± 6.24	−9.34 ± 7.58	−11.3 ± 6.84	−9.07 ± 8.17	−11.6 ± 9.19	−8.47 ± 7.49
Change *vs* MedLift *p*-value	—	—	<0.01	<0.01	0.05	<0.01	0.28	<0.01	0.56	<0.01
QoL *N* (paired)	155	930	97	564	67	393	22	170	25	155
Baseline (SD)	4.23 ± 1.19	4.19 ± 1.61	4.26 ± 1.18	4.10 ± 1.16	4.30 ± 1.10	4.11 ± 1.26	3.77 ± 1.07	4.15 ± 1.22	4.04 ± 1.14	3.90 ± 1.23
Follow-up (SD)	—	—	2.19 ± 1.62	2.26 ± 1.48	1.70 ± 1.26	2.36 ± 1.57	1.95 ± 1.46	2.29 ± 1.57	1.96 ± 1.31	2.27 ± 1.46
Change (SD)	0.00 ± 0.00	0.00 ± 0.00	−2.07 ± 1.87	−1.85 ± 1.73	−2.60 ± 1.52	−1.75 ± 1.70	−1.82 ± 1.50	−1.86 ± 1.79	−2.08 ± 1.96	−1.63 ± 1.62
Change *vs* MedLift *p*-value	—	—	<0.01	<0.01	0.07	<0.01	0.03	<0.01	0.06	<0.01
PVR *N* (paired)	114	850	82	606	48	357	19	152	17	147
Baseline (SD)	74.53 ± 68.61	71.19 ± 62.19	66.30 ± 60.64	70.79 ± 63.04	62.88 ± 56.47	73.38 ± 65.02	40.47 ± 56.34	90.84 ± 68.59	82.76 ± 72.04	78.35 ± 64.57
Follow-up (SD)	—	—	45.73 ± 54.84	59.87 ± 77.20	54.08 ± 67.88	64.48 ± 79.63	56.42 ± 83.56	71.75 ± 104.9	83.53 ± 85.14	67.75 ± 78.97
Change (SD)	0.00 ± 0.00	0.00 ± 0.00	−20.6 ± 60.16	−10.9 ± 86.31	−8.79 ± 63.74	−8.90 ± 84.27	15.95 ± 96.85	−19.1 ± 118.1	0.76 ± 70.97	−10.6 ± 82.73
Change *vs* MedLift *p*-value	—	—	0.6	0.14	0.44	0.25	0.25	0.77	0.22	0.12
Qmax *N* (paired)	53	322	15	100	6	99	3	23	1	42
Baseline (SD)	7.61 ± 2.12	8.35 ± 2.40	8.36 ± 2.07	8.26 ± 2.42	7.13 ± 1.48	8.55 ± 2.37	7.57 ± 3.61	8.55 ± 2.30	7.4	8.81 ± 2.17
Follow-up (SD)	—	—	11.99 ± 4.31	12.64 ± 5.83	10.42 ± 4.59	13.29 ± 6.18	7.97 ± 3.55	13.04 ± 4.43	14.5	11.94 ± 7.28
Change (SD)	0.00 ± 0.00	0.00 ± 0.00	3.63 ± 4.50	4.38 ± 6.27	3.28 ± 3.53	4.74 ± 6.07	0.40 ± 0.53	4.49 ± 4.39	7.1	3.13 ± 6.74
Change *vs* MedLift *p*-value	—	—	0.08	0.01	0.28	0.06	0.17	0.81	0.99	0.1

**Table 5.** Sexual Function and Satisfaction Outcomes over 12 Months Following Treatment with Prostatic Urethral Lift

	Baseline				1 Month				3 Months				6 Months				12 Months			
	MedLift	L.I.F.T.–LL	L.I.F.T.–sham	BPH–TURP	MedLift	L.I.F.T.–LL	L.I.F.T.–sham	BPH–TURP	MedLift	L.I.F.T.–LL	L.I.F.T.–sham	BPH–TURP	MedLift	L.I.F.T.–LL	L.I.F.T.–sham	BPH–TURP	MedLift	L.I.F.T.–LL	L.I.F.T.–sham	BPH–TURP
SHIM *N* (paired)	39	107	53	35	35	88	49	20	36	91	49	27	38	94	12	30	38	87	7	27
Baseline (SD)	17.23 ± 7.68	13 ± 8.4	15.72 ± 7.46	17.94 ± 5.47	17.29 ± 7.59	16.28 ± 7.112	16.29 ± 7.19	17.55 ± 6.17	17.47 ± 7.49	16.16 ± 7.02	15.98 ± 7.27	19.19 ± 5.01	17.63 ± 7.36	16.27 ± 7.01	19.25 ± 6.76	18.37 ± 5.35	17.24 ± 7.78	15.99 ± 7.14	20.14 ± 4.74	18.59 ± 5.38
Follow-up (SD)	—	—	—	—	18.60 ± 8.09	17.25 ± 7.63	16.61 ± 7.36	17.20 ± 7.32	18.72 ± 7.78	17.44 ± 7.58	17.24 ± 6.71	18.22 ± 6.53	17.26 ± 8.40	17.33 ± 7.63	19.67 ± 6.36	17.60 ± 6.47	18.42 ± 8.30	16.69 ± 7.76	21.00 ± 3.70	17.70 ± 6.26
Change (SD)	0.00 ± 0.00	0.00 ± 0.00	0.00 ± 0.00	0.00 ± 0.00	1.31 ± 3.64	.9 ± 5.43	0.33 ± 3.05	−0.35 ± 4.87	1.25 ± 4.48	1.27 ± 4.65	0.98 ± 3.91	−0.96 ± 5.03	−0.37 ± 6.09	1.06 ± 4.30	0.42 ± 2.11	−0.77 ± 4.58	1.18 ± 4.27	0.7 ± 5.12	0.86 ± 1.57	−0.89 ± 4.27
Change *vs* MedLift *p*-value	—	—	—	—	—	0.7	0.18	0.16	—	1	0.77	0.07	—	0.1	0.67	0.77	—	0.6	0.84	0.06
MSHQ EJD *N* (paired)	39	94	53	35	35	88	49	18	36	91	50	27	38	94	12	29	38	87	7	27
Baseline (SD)	9.36 ± 3.12	8.7 ± 3.2	8.81 ± 3.16	11.11 ± 2.35	9.20 ± 3.13	8.92 ± 3.08	9.00 ± 3.03	10.56 ± 2.45	9.39 ± 3.07	8.67 ± 3.09	8.76 ± 3.10	11.22 ± 2.44	9.45 ± 3.11	8.76 ± 3.23	10.00 ± 3.02	10.90 ± 2.26	9.42 ± 3.13	8.69 ± 3.26	9.57 ± 3.26	11.33 ± 2.04
Follow-up (SD)	—	—	—	—	11.43 ± 3.08	11.22 ± 3.30	10.71 ± 2.98	9.39 ± 4.24	11.25 ± 3.38	10.98 ± 3.16	10.48 ± 3.52	8.52 ± 3.92	11.21 ± 3.09	10.53 ± 3.29	10.67 ± 3.65	7.62 ± 3.40	11.45 ± 2.79	10.25 ± 3.16	10.71 ± 2.93	7.59 ± 3.72
Change (SD)	0.00 ± 0.00	0.00 ± 0.00	0.00 ± 0.00	0.00 ± 0.00	2.23 ± 2.45	2.30	1.71 ± 2.75	−1.17 ± 4.60	1.86 ± 2.85	2.31	1.72 ± 2.59	−2.70 ± 3.93	1.76 ± 2.83	1.78	0.67 ± 2.19	−3.28 ± 3.95	2.03 ± 2.79	1.56	1.14 ± 1.21	−3.74 ± 4.44
Change *vs* MedLift *p*-value	—	—	—	—	—	0.9	0.38	<0.01	—	0.4	0.82	<0.01	—	1.0	0.23	<0.01	—	0.4	0.42	<0.01

Expectedly, MedLift patients experienced more adverse events (30.3% *vs* 88.9%, *p* < 0.01) and higher rates of postprocedural catheterization (for subjects who failed voiding trials: 15.6% *vs* 3.0%, *p* < 0.01) compared with sham subjects. As previously published, perioperative adverse events were typically mild to moderate and transient and mean catheter duration was 1.2 days averaged over the total cohort.^10^ Compared with patients who underwent TURP, MedLift subjects experienced no high-severity adverse events (AEs) (0.0% *vs* 14.3%, *p* < 0.01), whereas five TURP patients reported serious AEs (blood clot in urine, urge incontinence, urethral stricture, bladder tamponade, and weak urinary stream); MedLift patients also experienced shorter catheter durations in comparison to TURP (1.24 days *vs* 2.2 days, *p* = 0.01). Retreatment rates were similar between MedLift and TURP patients (2.2% *vs* 5.7%, *p* = 0.4155).

### PUL for OML in large-scale retrospective study confirms control trials

PUL outcomes for treating OML were equivalent to those for treating LL in the RWR study. At baseline, RWR OML and LL groups were equivalent in terms of IPSS, QoL, Qmax, and age. Postprocedure IPSS, QoL, Qmax, and PVR were similar at 1, 6, and 12 months (Table 4). Overall AEs and nonstandard of care catheterization rates were also similar between RWR OML and LL groups.

Compared with the filtered RWR OML subjects, MedLift patients were younger and more symptomatic at baseline while baseline body mass index and prostate specific antigen (PSA) were equivalent (Table 2). Although the filtered RWR OML group retained significant baseline differences in comparison to MedLift, the cohorts were as closely matched as possible to include patients at 6- and 12-month time points from which to make meaningful comparisons.

Post-PUL, percent change IPSS, QoL, PVR, and Qmax were equivalent between MedLift and the filtered RWR OML group at 3, 6, and 12 months (Table 3 and Fig. 2A,B). Collectively, MedLift, RWR OML, and RWR LL subjects had similar catheter-free rates (20% *vs* 33.9% and 62.1%, *p* = 0.07) and RWR cohorts did not experience higher rates of overall AEs (66.7% *vs* 29.4% and 31.3%).

## Discussion

Median lobe obstruction has been estimated to occur in up to 20% of men diagnosed with BPH, although in the L.I.F.T. RCT only 5.3% subjects were excluded due to median lobe obstruction.^10–13^ Although this type of obstruction may not affect most BPH patients, population-based studies have shown that an OML can pose an increased risk for progression of clinical BPH and bladder outlet obstruction, prompting the inclusion of diagnostic morphology assessments in AUA guidelines.^6,14,15^ In general, watchful waiting and most BPH medical therapies are less effective at treating patients with OML, often necessitating surgical intervention.^17–20^ As such, the access to minimally invasive surgical options for this patient population is particularly pertinent.

The utility of PUL in treating OML was first demonstrated in the MedLift CCT, where median lobe patients were enrolled using the L.I.F.T. trial criteria to approximate LL patients in the L.I.F.T. RCT. In the MedLift CCT, no significant difference was seen at 1 year between OML and LL outcomes. Notably, PUL efficacy in patients with OML was independent of intravesical prostatic protrusion (IPP) severity at baseline (*p* = 0.7).^10,16^ This independence is likely due to median lobe displacement during PUL, changing the angle of bladder neck obstruction and preventing the median lobe from acting as a “ball valve” into the prostatic fossa. Durability is likely associated with displacement and the resulting ischemia, atrophy, and scarring that occurs postimplantation.^5,21^

In the current analysis, we find that PUL for OML indeed outperforms matched sham controls without OML in terms of symptom relief, QoL, and flow rate, while showing no relative negative change in sexual function. As one would expect, there was a higher postoperative catheterization rate for OML PUL, but as with LL patients the typical catheter duration was ∼1 day. At the 3-month comparator endpoint PUL for OML was more effective than sham and somewhat more effective in symptom relief and flow improvement than with LL. This is likely due to the dramatic effect displacement, regardless of IPP, can have on outcomes. Although the MedLift and BPH6 studies utilized different criteria scales to assess adverse events (CTCAE and Clavien–Dindo, respectively) a strength of PUL was that no patient experienced a high-severity AE compared with TURP (i.e., CTCAE grade 3 *vs* Clavien–Dindo 3b). Furthermore, as expected, the adverse event rate in MedLift was higher than sham, lower than TURP, and commensurate with other MISTs.^22–24^

Comparing PUL for OML with TURP provides interesting insights. TURP, being a full cavitation of the prostatic fossa, generates higher flow rates, but it is interesting that the high flow did not translate into greater QoL. The trajectory of symptom improvement and patient satisfaction over the first year is particularly meaningful. PUL for OML patients improve and indeed are largely satisfied by 1 month, while TURP patients only catch up to the PUL patients by 6 to 12 months, showing no difference thereafter. Erectile function was maintained in both groups. While not surprising for PUL, the lack of degradation that was seen following TURP was perhaps due to rigorous baseline sexual function screening, TURP technique, or insufficient sample size to detect a low-level occurrence. Ejaculatory function is maintained for PUL patients, which is a marked difference to TURP. While TURP efficacy remains a gold standard for BPH, the considerably more rapid improvement, easier postoperative recovery, and preservation of ejaculatory function are likely important factors in OML men choosing PUL as a therapeutic choice.

The RWR study of PUL outcomes is the largest, most comprehensive investigation of a MIST for BPH. This comparative analysis expands on previously published clinical trial results demonstrating PUL's safety and efficacy in BPH subjects with OMLs. In this study, OML patients constituted 11.7% (244/2078). Patients treated with PUL outside of controlled trials appear to be modestly less symptomatic than in the typical controlled trial, which sets a minimum baseline IPSS at 13.^7,22,25^ Although published analyses of the RWR database have found the patients to be less symptomatic at baseline,^25^ when the database was filtered using MedLift enrollment criteria, a MedLift-like cohort emerged with mostly consistent baseline variables on which to build comparisons, strengthening the analytical approach. Despite the IPP measurement not widely available for extraction as part of the retrospective real-world database, it is encouraging that symptom improvement in the real-world largely reflected results from the controlled MedLift study. One can deduce that with over 200 real-world OML patients in the database, IPP and OML anatomical variability existed, however additional investigation in this arena will further aid patient selection.

Outcomes for PUL treating OML were largely equivalent in CCT and real-world studies, with similar percent improvement in IPSS, QoL, and Qmax scores through 12 months. Catheterization rates and duration were also comparable. Mild-to-moderate adverse events are more prevalent in the CCT, which could be a result of closer scrutiny of a controlled study follow-up or a reflection of refined technique with more experience. While large-scale real-world clinical data are not considered by many academic guidelines, it nonetheless presents an important test as to whether a treatment performs in the real world as well as it performed in highly controlled clinical studies. The data from over 3000 patients across myriad sites indeed indicate that PUL is as effective in treating OML and LL disease in the ambulatory setting as it was shown to be in controlled studies.

Although this study draws conclusions from patient populations across nonconcomitant studies, the trials pooled for this comparative analysis encompass PUL treatment spanning 2011 through 2019. A critical foundation of this analysis was that baseline demographics were similar for RCT and CCT groups, except for baseline Qmax, which was higher for TURP controls *vs* MedLift subjects. To date, PUL is the most well-studied MIST for BPH. The ability to evaluate results across multiple clinical controlled studies (including two RCTs) and compare results to a large real-world study is a key strength of the technology.

## Conclusion

In this first detailed analysis of patients with OML treated with PUL in CCT and real-world settings compared with subjects treated with TURP or sham in RCTs not selecting for men with OML, we show that PUL is superior to TURP within the first 3 months following treatment, with better symptom improvement and patient experience and no serious adverse events. Outcomes from the large RWR study of PUL further support the effectiveness of the procedure, demonstrating similar symptom improvement and catheter-free rates with no elevation in overall adverse event rates in the real-world setting.

The clinical value of this study is achieved through total access to a large database of outcomes from industry-sponsored studies. By comparing results from randomized, clinical controlled, and large real-world studies, we were able to achieve a robust view of the safety and effectiveness of PUL in treating OML and LL obstruction. While academic guidelines often limit their analyses to early randomized studies for regulatory approval, we believe there is important information to be gleaned from comparing these results to present-day results in our clinics. This analysis supports the use of the PUL for treatment of BPH in prostates with or without OMLs.

## Abbreviations Used

AEs: adverse events
AUA: American Urological Association
BPH: benign prostatic hyperplasia
BPHII: benign prostatic hyperplasia impact index
CCT: controlled clinical trial
CI: confidence intervals
EAU: European Association of Urology
EJD: Ejaculatory Dysfunction
FDA: U.S. Food and Drug Administration
IPP: intravesical prostatic protrusion
IPSS: international Prostate Symptoms Score
L.I.F.T.: Luminal Improvement Following Prostatic Tissue Approximation for the Treatment of Lower Urinary Tract Symptoms
LL: lateral lobe
MIST: minimally invasive surgical therapy
MSHQ: Male Sexual Health Questionnaire
OML: obstructive median lobe
PUL: prostatic urethral lift
PVR: postvoid residual
Qmax: peak flow rate
QoL: quality of life
RCT: randomized controlled trial
RWR: real-world retrospective
SHIM: Sexual Health Inventory for Men
TURP: transurethral resection of the prostate

## References

[1] Rijo E, Hindley R, Tabatabaei S, et al. Minimally invasive surgery for benign prostatic obstruction: New insights and future technical standards. Curr Opin Urol 2021;31(5):461–467; doi: 10.1097/MOU.0000000000000918. 34231545

[2] Mahon JT, Welliver C. National trends in the management of lower urinary tract symptoms associated with benign prostatic hyperplasia. Curr Urol Rep 2020;21(12):63; doi: 10.1007/s11934-020-01014-w. 33210174

[3] Garden E, Tomer N, Al-Alao O, et al. MP28-09 contemporary trends in utilization and Medicare reimbursement for ambulatory BPH procedures (2014–2018). J Urol 2021;206(Supplement 3):e484. https://www.auajournals.org/doi/pdf/10.1097/JU.0000000000002025.09

[4] Dalimov Z, Hamann H, Alavi-Dunn Nicole, et al. PD29-09 trends in minimally invasive surgical therapies for benign prostatic hyperplasia: Treatment substitution or treatment expansion effect by prostatic urethral lift? J Urol 2020;203(Supplement 4S):e621. https://www.auajournals.org/doi/pdf/10.1097/JU.0000000000000893.09

[5] Roehrborn CG, Chin PT, Woo HH. The UroLift implant: Mechanism behind rapid and durable relief from prostatic obstruction. Prostate Cancer Prostatic Dis 2022;25(1):79–85; doi: 10.1038/s41391-021-00434-0. 34363010 PMC9018420

[6] American Urological Association (AUA). Guideline. Benign Prostatic Hyperplasia: Surgical Management of Benign Prostatic Hyperplasia/Lower Urinary Tract Symptoms (2021). Available from: https://www.auanet.org/guidelines/guidelines/benign-prostatic-hyperplasia-(bph)-guideline [Last accessed: November 10, 2021].

[7] Roehrborn CG, Barkin J, Gange SN, et al. Five year results of the prospective randomized controlled prostatic urethral L.I.F.T. study. Can J Urol 2017;24(3):8802–8813. 28646935

[8] Roehrborn CG, Rukstalis DB. Prostatic urethral lift versus medical therapy: Examining the impact on sexual function in men with benign prostatic hyperplasia. Eur Urol Focus 2022;8(1):217–227; doi: 10.1016/j.euf.2020.12.013. 33436276

[9] European Association of Urology (EAU). Guideline. Management of Non-neurogenic Male LUTS. Available from: https://uroweb.org/guideline/treatment-of-non-neurogenic-male-luts/ [Last accessed: May 9, 2022].

[10] Rukstalis D, Grier D, Stroup SP, et al. Prostatic urethral lift (PUL) for obstructive median lobes: 12 month results of the MedLift study. Prostate Cancer Prostatic Dis 2019;22(3):411–419; doi: 10.1038/s41391-018-0118-x. 30542055 PMC6760566

[11] Wei JT, Calhoun E, Jacobsen SJ. Urologic diseases in America project: Benign prostatic hyperplasia. J Urol 2008;179(5 Suppl):S75–S80; doi: 10.1016/j.juro.2008.03.141. 18405761 PMC11160437

[12] Berry SJ, Coffey DS, Walsh PC, et al. The development of human benign prostatic hyperplasia with age. J Urol 1984;132(3):474–479; doi: 10.1016/s0022-5347(17)49698-4. 6206240

[13] Roehrborn CG, Gange SN, Shore ND, et al. The prostatic urethral lift for the treatment of lower urinary tract symptoms associated with prostate enlargement due to benign prostatic hyperplasia: The L.I.F.T. study. J Urol 2013;190(6):2161–2167; doi: 10.1016/j.juro.2013.05.116. 23764081

[14] Kuo TL, Teo JS, Foo KT. The role of intravesical prostatic protrusion (IPP) in the evaluation and treatment of bladder outlet obstruction (BOO). Neurourol Urodyn 2016;35(4):535–537; doi: 10.1002/nau.22741. 25727301

[15] Chia SJ, Heng CT, Chan SP, et al. Correlation of intravesical prostatic protrusion with bladder outlet obstruction. BJU Int 2003;91(4):371–374; doi: 10.1046/j.1464-410x.2003.04088.x. 12603417

[16] Yoshida T, Kinoshita H, Yoshida K, et al. Intravesical prostatic protrusion as a predicting factor for the adverse clinical outcome in patients with symptomatic benign prostatic enlargement treated with dutasteride. Urology 2016;91:154–157; doi: 10.1016/j.urology.2016.01.009. 26826590

[17] Kalkanli A, Tandogdu Z, Aydin M, et al. Intravesical prostatic protrusion: A potential marker of alpha-blocker treatment success in patients with benign prostatic enlargement. Urology 2016;88:161–165; doi: 10.1016/j.urology.2015.11.029. 26680245

[18] Eze BU, Ani CO, Mbaeri TU. Is intravesical prostatic protrusion associated with more complications in benign prostatic hyperplasia patients? Low Urin Tract Symptoms 2021;13(4):468–474; doi: 10.1111/luts.12394. 34080315

[19] Gandhi J, Weissbart SJ, Kim AN, et al. Clinical considerations for intravesical prostatic protrusion in the evaluation and management of bladder outlet obstruction secondary to benign prostatic hyperplasia. Curr Urol 2018;12(1):6–12; doi: 10.1159/000447224. 30374274 PMC6198776

[20] Hirayama K, Masui K, Hamada A, et al. Evaluation of intravesical prostatic protrusion as a predictor of dutasteride-resistant lower urinary tract symptoms/benign prostatic enlargement with a high likelihood of surgical intervention. Urology 2015;86(3):565–569; doi: 10.1016/j.urology.2015.05.021. 26199172

[21] Rabinowitz MJ, Alam R, Liu JL, et al. Prostatic urethral lift in patients with obstructive median lobes: A single surgeon experience at an academic center. Urology 2021;154:237–242; doi: 10.1016/j.urology.2021.01.025. 33493510

[22] McVary KT, Rogers T, Roehrborn CG. Rezūm water vapor thermal therapy for lower urinary tract symptoms associated with benign prostatic hyperplasia: 4-Year results from randomized controlled study. Urology 2019;126:171–179; doi: 10.1016/j.urology.2018.12.041. 30677455

[23] Ottaiano N, Shelton T, Sanekommu G, et al. Surgical complications in the management of benign prostatic hyperplasia treatment. Curr Urol Rep 2022;23(5):83–92; doi: 10.1007/s11934-022-01091-z. 35262855

[24] Tanneru K, Jazayeri SB, Alam MU, et al. An indirect comparison of newer minimally invasive treatments for benign prostatic hyperplasia: A network meta-analysis model. J Endourol 2021;35(4):409–416; doi: 10.1089/end.2020.0739. 32962442

[25] Eure G, Gange S, Walter P, et al. Real-world evidence of prostatic urethral lift confirms pivotal clinical study results: 2-Year outcomes of a retrospective multicenter study. J Endourol 2019;33(7):576–584; doi: 10.1089/end.2019.0167. 31115257 PMC6657298

